# The neurometabolic profiles of GABA and Glutamate as revealed by proton magnetic resonance spectroscopy in type 1 and type 2 diabetes

**DOI:** 10.1371/journal.pone.0240907

**Published:** 2020-10-29

**Authors:** Otília C. d’Almeida, Ines R. Violante, Bruno Quendera, Carolina Moreno, Leonor Gomes, Miguel Castelo-Branco

**Affiliations:** 1 Faculty of Medicine, University of Coimbra, Coimbra, Portugal; 2 CiBIT, Coimbra Institute for Biomedical Imaging and Translational Research, Institute of Nuclear Sciences Applied to Health (ICNAS), University of Coimbra, Coimbra, Portugal; 3 School of Psychology, Faculty of Health and Medical Sciences, University of Surrey, Guildford, United Kingdom; 4 Department of Endocrinology, Coimbra University and Hospital Centre (CHUC), Coimbra, Portugal; McLean Hospital, UNITED STATES

## Abstract

Glucose metabolism is pivotal for energy and neurotransmitter synthesis and homeostasis, particularly in Glutamate and GABA systems. In turn, the stringent control of inhibitory/excitatory tonus is known to be relevant in neuropsychiatric conditions. Glutamatergic neurotransmission dominates excitatory synaptic functions and is involved in plasticity and excitotoxicity. GABAergic neurochemistry underlies inhibition and predicts impaired psychophysical function in diabetes. It has also been associated with cognitive decline in people with diabetes. Still, the relation between metabolic homeostasis and neurotransmission remains elusive.

Two 3T proton MR spectroscopy studies were independently conducted in the occipital cortex to provide insight into inhibitory/excitatory homeostasis (GABA/Glutamate) and to evaluate the impact of chronic metabolic control on the levels and regulation (as assessed by regression slopes) of the two main neurotransmitters of the CNS in type 2 diabetes (T2DM) and type 1 diabetes (T1DM).

Compared to controls, participants with T2DM showed significantly lower Glutamate, and also GABA. Nevertheless, higher levels of GABA/Glx (Glutamate+Glutamine), and lower levels of Glutamate were associated with poor metabolic control in participants with T2DM. Importantly, the relationship between GABA/Glx and HbA_1c_ found in T2DM supports a relationship between inhibitory/excitatory balance and metabolic control. Interestingly, this neurometabolic profile was undetected in T1DM. In this condition we found strong evidence for alterations in MRS surrogate measures of neuroinflammation (myo-Inositol), positively related to chronic metabolic control.

Our results suggest a role for Glutamate as a global marker of T2DM and a sensitive marker of glycemic status. GABA/Glx may provide a signature of cortical metabolic state in poorly controlled patients as assessed by HbA_1c_ levels, which indicate long-term blood Glucose control. These findings are consistent with an interplay between abnormal neurotransmission and metabolic control in particular in type 2 diabetes thereby revealing dissimilar contributions to the pathophysiology of neural dysfunction in both types of diabetes.

## Introduction

Normal brain function relies on the stringent control of the levels of the main inhibitory (GABA) and excitatory (Glutamate) neurotransmitters. The dysregulation of the inhibitory/excitatory (I/E) balance in local circuitry and neural networks has been suggested to play a role in the pathophysiology of a broad range of neurodevelopmental and neuropsychiatric disorders [1,2].

GABA and Glutamate pools are commonly compartmentalized into neurotransmitter and metabolic parcels [3,4] having dual roles. These key metabolites are dependent on brain Glucose [5] and synaptic signaling strongly relies on the interactions between astrocytes and neurons through the GABA-Glutamate-Glutamine shuttle [6–8]. This cycling is pivotal to the production, reuse and metabolism of both GABA and Glutamate as well as in the context of energy production [6,7]. Under normal conditions, Glutamate has a high flux rate and is closely coupled with the high-energy demands for brain functioning by contributing to the replenishment of Kreb’s cycle substrates namely in the synthesis of both N-acetylaspartate and alpha-ketoglutarate [9]. Since the neural tissue relies mainly on glucose content to fulfill the high energetic demands, the brain becomes a vulnerable target of damage in conditions characterized by impaired metabolic activity [10].

Diabetes Mellitus is a chronic disease estimated to affect nearly 415 million adults worldwide [11], characterized by an abnormal increase of blood Glucose (hyperglycemia) caused by lack of insulin production (type 1 diabetes, T1DM) or by predominant insulinoresistance (type 2 diabetes, T2DM) [12]. It is well known that the diabetic state alters cerebral structure, vascularization and metabolism [10,13,14]. Therefore, the extent to which long-term, chronic fluctuations in Glucose levels have repercussions in neurotransmission is a relevant question concerning the brain complications of diabetes. Yet existent studies investigating neurochemistry in diabetes are still scarce and heterogeneous and this relationship remains obscure [15,16].

Proton Magnetic Resonance Spectroscopy (^1^H-MRS) is a sensitive *in vivo* technique that allows to quantify several biomolecules such as N-acetylaspartate- (tNAA) and creatine-containing compounds (tCr) and to indirectly assess synaptic neurotransmission through a reliable estimation of Glutamate and GABA that are ubiquitously expressed throughout the cortex [17,18]. Regarding inhibitory neurotransmission, we previously found that impaired visual function in T2DM could be related to altered cortical neurochemistry within the GABAergic system (i.e. higher GABA levels have a deleterious effect on visual brain function) [19]. Also, van Bussel *et al*. [47] found that higher occipital GABA was correlated with T2DM and cognitive impairment, which is consistent with our previous findings. In fact, there is strong evidence supporting a pathophysiological link between T2DM, dementia and Alzheimer’s disease, possibly related to glycemic control and insulin dysregulation [20,21]. Also, randomized controlled trials (RCTs) have shown that glycemic control is tightly associated to the microvascular and neurological complications found in diabetes [22].

Additionally, a previous study applying ^1^H-MRS in diabetes suggested the existence of alterations in inhibitory (GABAergic) and possibly also excitatory (indirectly assessed by the levels of the Glutamate+Glutamine pool (Glx)) neurotransmission in several brain regions [23]. By contrast, other studies have not replicated changes in the GABAergic neurotransmitter system, but instead in NAA-containing compounds levels in T2DM [24,25] or in both Glutamatergic pools and NAA in type 1 diabetes [26] suggesting that neurometabolic patterns may differ across conditions and even disease states.

In this work we intended to evaluate the I/E balance in T2DM and T1DM groups, inferred by the quantification of GABA and Glutamate through ^1^H-MRS and by comparing each to independent age-matched control groups. The main goal of our study was to evaluate the hypothesis that neurotransmission is associated with long-term metabolic control, evaluated by glycated hemoglobin (HbA_1c_) levels.

## Materials and methods

### Participants

We performed two independent studies with diabetes mellitus patients, diagnosed according to the current WHO criteria and recruited from the Endocrinology Department of Coimbra’s Hospital and University Centre. In one study we have studied one cohort of 26 type 2 diabetes patients (Study A, 42 participants in total) and in the other, we have studied 10 type 1 diabetes patients (Study B, 26 participants). Due to the age range of the patients in each of the diabetes groups, we have recruited two independent age-matched control groups, both recruited from the local community. Participant characteristics are given in Table 1.

**Table 1:** General clinical-demographic characterization of the cohorts under study.

**Study A–Type 2 diabetes mellitus**			
**Measure**	**T2DM**	**Ctrl2**	**Statistic**
N	26	16	
Age^a^ (y)	62.0 ± 8.45	63.3 ± 9.16	*t*_40_ = -0.46; *p* = 0.648
Age range (min:max, y)	48:76	48:79	
Gender (M:F, %)	57.7: 42.3	50.0: 50.0	χ^2^_1_ = 0.24; *p* = 0.627
BMI^a^ (kg/m^2^)	29.9 ± 4.73 (N = 25)	25.0 ± 3.60 (N = 15)	t_38_ = 3.43; *p* = 0.001
Disease duration^a^ (y)	15.8 ± 5.21 (N = 25)	--	
HbA_1c_ level^a^ (%)	7.9 ± 1.26	--	
HbA_1c_ level^a^ (mmol/mol)	62.4 ± 13.80	--	
Major MRI alterations	no	no	
Tissue fraction of the ^1^H-MRS voxel			
fGM^a^ (%)	50.7 ± 4.32	51.9 ± 4.30	*t*_40_ = -0.90, *p* = 0.375
fWM^a^ (%)	34.8 ± 4.94	32.0 ± 5.32	*t*_40_ = 1.70, *p* = 0.097
fCSF^a^ (%)	14.5 ± 5.54	16.1 ± 5.31	*t*_40_ = -0.88, *p* = 0.386
**Study B–Type 1 diabetes mellitus**			
**Measure**	**T1DM**	**Ctrl1**	**Statistic**
N	10	16	
Age^a^ (y)	35.2 ± 4.87	33.1 ± 7.58	*Z* = -0.79; *p* = 0.452
Age range (min:max, y)	31:46	24:47	
Gender (M:F, %)	70.0: 30.0	56.3: 43.8	*p* = 0.683^b^
BMI^a^ (kg/m^2^)	26.1 ± 3.68 (N = 8)	24.7 ± 3.87	*Z* = -0.98; *p* = 0.350
Disease duration^a^ (y)	24.3 ± 3.28 (N = 9)	--	
HbA_1c_ level^a^ (%)	8.3 ± 1.79 (N = 7)	--	
HbA_1c_ level^a^ (mmol/mol)	66.9 ± 19.79 (N = 7)	--	
Major MRI alterations	no	no	
Tissue fraction of the ^1^H-MRS voxel			
fGM^a^ (%)	54.1 ± 5.14	57.6 ± 2.48	*Z* = -1.85, *p* = 0.068
fWM^a^ (%)	32.9 ± 3.48	32.0 ± 3.41	*Z* = -0.74, *p* = 0.484
fCSF^a^ (%)	13.0 ± 3.98	10.4 ± 3.08	*Z* = -1.66, *p* = 0.097

BMI, Body mass index; Ctrl1, Control group for T1DM group; Ctrl2, Control group for T2DM group; T1DM, Type 1 diabetes; T2DM, Type 2 diabetes.

^a^ Values are represented as mean ± standard deviation (SD) for each group.

^b^ Chi-square Fisher’s Exact test.

Exclusion criteria for all groups comprehended the presence of cataract, glaucoma, any other eye disease, surgery, or treatment within a period of 6-months and severe nonproliferative (ETDRS level > 35) or proliferative retinopathy. Pregnant or lactating women, participants with chronic or severe kidney disease or acute kidney injury, severe cardiovascular problems, with cardiac pacemaker or metal implants in the body were also excluded. All participants reported no history of neurological or psychiatric disorders and had no neurovascular and structural pathologic alterations as assessed by an experienced neuroradiologist. Control participants had no history of diabetes.

In order to evaluate patients’ metabolic control level, blood samples were collected for analysis of glycated hemoglobin (HbA_1c_). This was assessed by high-performance liquid chromatography (Variant II, Bio-Rad). In the T2DM group, 19 participants were taking oral antidiabetic agents (OAD) and 6 insulin alone or in conjunction with OAD. One T2DM participant had no reference of the prescribed medication.

The study was reviewed and approved by the Ethics Commission of the Faculty of Medicine of the University of Coimbra and followed the tenets of the Declaration of Helsinki. Written informed consent was obtained from all participants.

### MRI data acquisition

MRI acquisitions were conducted on a 3T MRI scanner (Siemens Magnetom 3T TimTrio, Erlangen, Germany) at the Institute of Nuclear Sciences Applied to Health (ICNAS, University of Coimbra) using a 12-channel birdcage head coil. Each participant underwent conventional high-resolution T1-weighted three-dimensional Magnetization Prepared Rapid Acquisition Gradient Echo (MPRAGE) sequence [repetition time (TR) 2530 ms, echo time (TE) 3.42 ms, inversion time (TI) 1100 ms, flip angle (FA) 7°, field of view (FOV) 256 × 256 mm^2^, yielding 176 slices with 1 × 1 × 1 mm^3^ voxel size].

**Fig 1:**
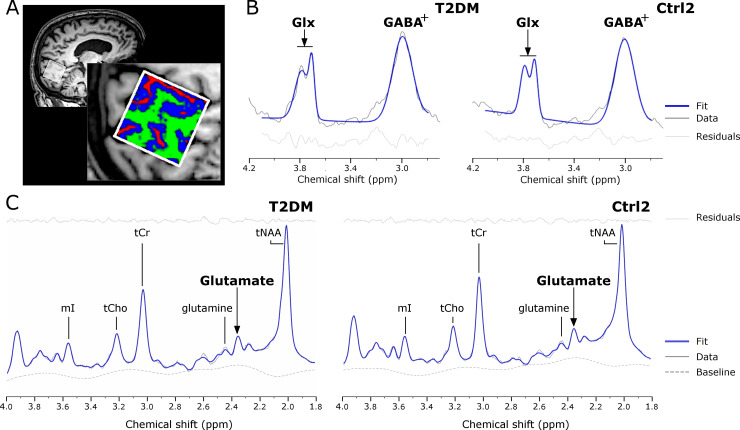
Spectroscopic acquisition and data processing. Sagittal view of a (A) representative magnetic resonance spectroscopy voxel acquired in the gray matter rich occipital lobe of a T2DM patient (male, 69 y). The magnified inset shows a sagittal view of the MRS voxel segmented into the three main tissue types, gray matter (blue), white matter (green), and cerebrospinal fluid (red). Two different ^1^H-MRS acquisition sequences were used: (B) MEGA-PRESS, to estimate γ-aminobutyric acid (GABA^+^) and Glx levels (analyzed through Gannet) and (C) PRESS, to estimate particularly Glutamate (analyzed through LCModel) and other metabolites. Both MEGA-PRESS and PRESS spectroscopy data are from a T2DM patient (female, 64 years) and an age- and gender-matched healthy control (Ctrl2, female, 64 years). In (B), it is represented the post-aligned edited data (dark gray solid line), the Gannet fitted model curve (bold blue solid line) and the residuals (light gray solid line, at the bottom) and in (C), the processed PRESS data (gray solid line), the LCModel fitted spectrum (bold blue solid line), the residuals (light gray solid line, on top) and the baseline (light gray dashed line) are shown.

MRS spectra were acquired in a 3 x 3 x 3 cm^3^ voxel positioned medially in the occipital cortex (Fig 1A) to make a compromise between voxel localization and signal-to-noise ratio [27]. The volume-of-interest was specifically chosen to cover a high content of gray matter (Table 1). GABA and Glx levels were measured in all participants using the MEshcher-GArwood Point RESolved Spectroscopy (MEGA-PRESS) sequence [28] [TR 1500 ms, TE 68 ms, FA 90°, 392 averages, 1024 data points]. Editing frequency-selective inversion pulses were applied to the GABA-C3 resonance at 1.9 ppm (refocused ‘on resonance’) and 7.5 ppm (non-refocused ‘off resonance’) during odd and even number acquisitions, respectively. Since most peaks in the spectrum are undisturbed by the applied editing pulses, subtracting ‘on’ and ‘off’ spectra remove these peaks and retains the GABA and Glutamate+Glutamine (Glx) peaks from the spectrum. To calculate water-scaled concentrations, MEGA-PRESS spectra without the suppression of the water signal (32 averages) were acquired in the same location.

In addition, participants were submitted to a Point RESolved Spectroscopy (PRESS) sequence acquisition [TR 2000 ms, TE 35 ms, FA 90°, 160 averages, 1024 data points] to estimate Glutamate (independently from the Glutamine peak) and evaluate other possibly relevant metabolites such as N-Acetylaspartate- (tNAA), Creatine- (tCr) and Choline- (tCho) containing compounds, Glutamine, myo-Inositol (mI) and reduced Glutathione (GSH). It is important to emphasize that PRESS spectra with unsuppressed water signal (16 averages) were also acquired to estimate absolute metabolite concentrations.

### Data analysis

In order to quantify spectral data, it is usual to estimate metabolites ratios or absolute concentrations [29]. The most common internal reference for metabolites ratios is total creatine (tCr, as the pool of Creatine and Phosphocreatine) to correct for several experimental conditions and methodological differences. However, there is the assumption that the tCr signal is stable along tissues and disease states, development, or aging. This may not always be the case [30,31], and water reference has becoming preferable to use instead, allowing to estimate absolute values, allied to automatic tissue fractions segmentation algorithms [32] that promote smaller coefficients of variation. Therefore, we opted to analyze absolute estimates, in institutional units (i.u.). Nonetheless, since there were no significant differences of tCr levels between groups, estimated through PRESS, we opted to replicate some analyses by using tCr as internal reference.

MEGA-PRESS data were analyzed using Gannet GABA-MRS Analysis Tool [33] version 3.1.4 for MATLAB (R2020a, TheMathWorks, USA) to quantify GABA and Glx relative to water content (in institutional units, i.u.). All spectra were visually inspected and GABA or Glx data with an associated GABA^+^_error to water_ (combination of GABA and water fit errors) or Glx_error to water_ (combination of Glx and water fit errors) higher than 15%, respectively, were discarded from analysis. Mean±SD for GABA^+^_error to water_ was 4±0.9% for Study A and 4±1.9% for Study B and for Glx_error to water_ was 5±1.5% for Study A and 6±2.4% (N = 25) for Study B. A 3 Hz exponential line broadening was applied to all spectra prior to the Fast Fourier Transform of the time resolved data. After frequency and phase correction an edited difference spectrum was generated for each dataset. A nonlinear least-squares fitting was used to integrate the ~3.00 ppm GABA and the ~3.75 ppm Glx peaks from a three-Gaussian function with a nonlinear baseline applied in the difference spectrum fitted between 2.79 and 4.10 ppm (Fig 1B). The creatine signal was modeled from the OFF spectrum by a Lorentzian function and the unsuppressed water peak estimated from the OFF unsuppressed water spectrum as a mixed Gaussian-Lorentzian model. GABA signal will herein be referred as GABA^+^ to account for the possible contribution of Homocarnosine and Macromolecule signals [33]. Since GABA and Glx concentrations are highly dependent on the tissue composition [34,35] and GABA- and Glutamatergic activity occur mostly in the GM, lesser in the WM and is almost negligible in the CSF, we corrected its concentration (GABA^+^_corr_ and Glx_corr_) for voxel tissue composition within Gannet pipeline based on Harris *et al*. method [36]. The fractions of gray matter (GM), white matter (WM) and cerebrospinal fluid (CSF) enclosed in the acquired voxel were estimated from the anatomical T1-weighted images (Table 1, Fig 1A) using the coregistration and segmentation functions introduced in the Gannet toolkit relying on SPM12 toolbox (Wellcome Trust Centre for Neuroimaging, Institute of Neurology, UCL, London, UK, http://www.fil.ion.ucl.ac.uk/spm/) for MATLAB (R2020a, TheMathWorks, USA).

Post-processing and quantification of PRESS data was performed with LCModel version 6.3 [37]. The *in vivo* spectra were analyzed as a linear combination of prior knowledge *in vitro* standard basis dataset acquired with a PRESS sequence with TE 35 ms in a 3T scanner as in our study. Eddy-current correction and water scaling were performed enabling the estimation of absolute concentrations presented in institutional units (i.u.), approximating mmol per Kg wet weight. Spectra were analyzed between chemical shifts of 1.8 and 4 ppm to reduce major lipid and macromolecules artifacts on the filling baseline (Fig 1C). Only metabolites with Crámer-Rao Lower Bounds (CRLB) lower than 20% were considered for statistical analysis [37] to exclude poorly fitted data. Other metabolites were briefly inspected, as a secondary analysis: total NAA (tNAA, as the pool of N-Acetylaspartate and N-Acetylaspartylglutamate) and Choline-containing compounds (tCho, as the pool of Glycerophosphocholine and Phosphocholine), mI and Glutamine. PRESS-estimated Glucose levels were also assessed in the T2DM (N = 14, CRLB = 15±3.4%) and T1DM (N = 7, CRLB = 10±2.5%) cohorts. Partial volume correction for CSF fraction was performed automatically during the model fitting (http://s-provencher.com/pub/LCModel/manual/manual.pdf) using the equation described by Ernst *et al*. [38] that estimates a water content factor based on the fact that metabolites are mostly concentrated in the GM and WM. The values used to compute the correction factor were the voxel tissue composition fractions estimated from Gannet.

### Statistical analysis

All statistical analyses were carried out using IBM SPSS Statistics (version 24.0, IBM Corp., Armonk, NY, USA). Due to spectra quality constraints, one full PRESS spectrum of a T2DM individual was excluded from analysis. Metabolite variables that did not meet quality criteria were excluded from the analysis with a pairwise approach. Data normality assumption was verified for each clinical-demographic variable (age, disease duration, HbA_1c_ levels and BMI) and for metabolite estimates (GABA^+^_corr_, Glx, Glutamate) with the Shapiro-Wilk test. In Study A, comparisons between T2DM the respective age-matched control group (Ctrl2) relied on parametric independent samples t-tests or Mann-Whitney U tests when data did not meet normality assumptions. In Study B, non-parametric between-group comparisons were directly performed between T1DM and Ctrl1. Secondary *post hoc* analyses were also conducted between groups for other metabolite levels (tCr, Glutamine, tNAA, mI, tCho, GSH, and respective ratios to tCr). Effect sizes where calculated for main hypothesis testing (GABA^+^_corr_, Glx_corr_ and Glutamate) using Cohen’s *d*.

Further analysis was done within each diabetes group to evaluate the relation of inhibitory/excitatory balance with chronic peripheric (HbA_1c_) and acute central (PRESS-estimated Glucose) metabolic control. Linear regression analyses were performed to estimate the equation of the adjustment line and the linear correlation coefficient between HbA_1c_ and PRESS-estimated Glucose levels with GABA^+^_corr_, Glutamate and the GABA^+^_corr_/Glutamate ratio. The linear relation between chronic and acute metabolic control was assessed through linear regression fitting between HbA_1c_ and PRESS-estimated Glucose levels. In exploratory analysis, Spearman (*ρ*) or Pearson (*r*_*P*_) correlation analyses were applied to evaluate possible correlations between PRESS-estimated Glucose levels and neurotransmitters levels.

Spearman (*ρ*) correlation analyses were performed within the T2DM group and clinical-demographic variables (age, disease duration and BMI) in T2DM group. Two-tailed hypothesis testing was performed at a 0.05 significance level.

## Results

### Study A–Type 2 diabetes mellitus

#### Glutamate is a potential biomarker of disease in type 2 diabetes patients

**Fig 2:**
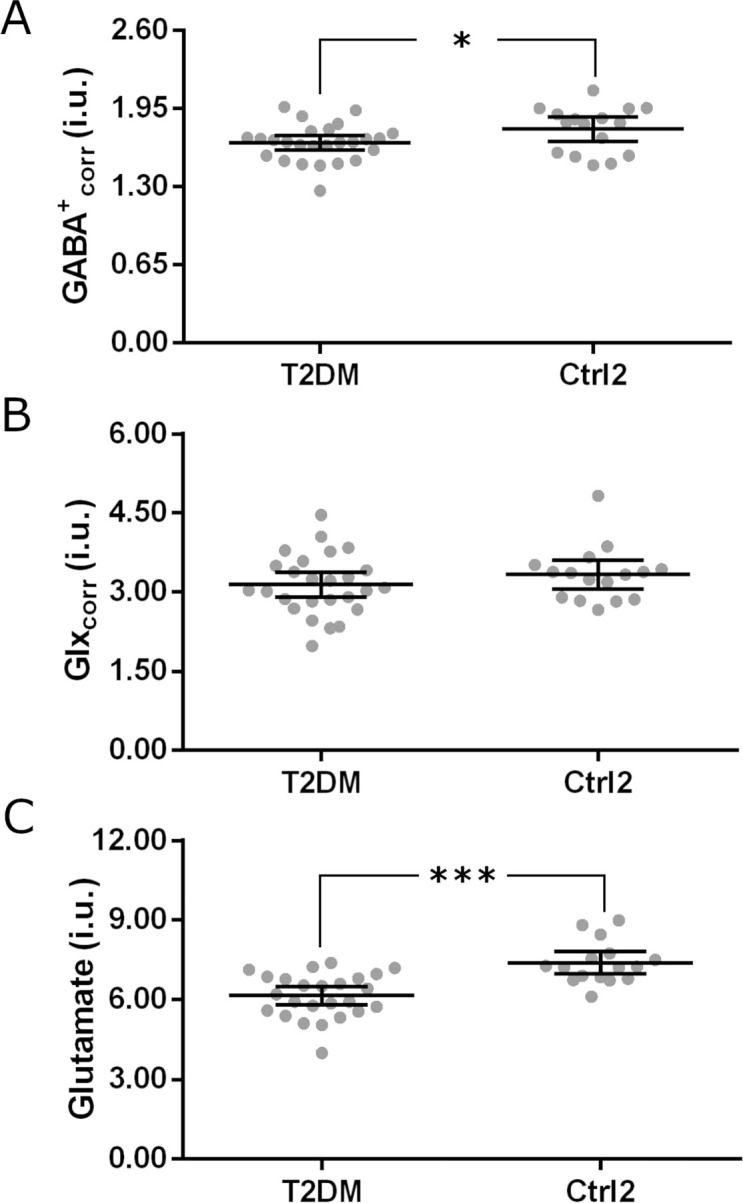
Metabolite levels in type 2 diabetes (T2DM) and age-matched healthy control (Ctrl2) groups were estimated through ^1^H-MRS. (A) GABA^+^_corr_ (i.u., institutional units) and (B) Glx_corr_ (i.u.) concentrations were estimated from MEGA-PRESS data and (C) Glutamate levels (i.u.) were estimated from PRESS data. The average level of GABA^+^_corr_ and Glutamate were significantly lower in T2DM group compared to Ctrl2 group. Graphs depict individual values and the mean (horizontal bar). Error bars represent 95% CI values. *p≤ 0.05,***p≤ 0.001.

In order to ascertain the putative differences on inhibitory and excitatory tonus, the levels of GABA and Glx between T2DM patients and the age-matched control (Ctrl2) group were evaluated (Fig 2A and 2B). A significant statistical difference was found for GABA^+^_corr_ concentration (*t*_40_ = -2.15; *p* = 0.038; GABA^+^_error to water_: T2DM = 4±0.9%, Ctrl2 = 3±0.9%; Cohen’s *d* = 0.68) with lower levels in T2DM group, yet no significant difference was found for Glx_corr_ levels between groups (*N* = 42, *Z* = -0.91, *p* = 0.365; Glx_error to water_: T2DM = 5±1.7%, Ctrl2 = 5±1.3%; Cohen’s *d* = 0.28). Since Glx reflects a mixed signal of Glutamate and Glutamine, we compared Glutamate levels estimated from PRESS data. A significant statistical difference was found (*t*_39_ = -4.70; *p* = 3.25x10^-5^; CRLB: T2DM = 9±2.4%, Ctrl2 = 8±1.1%; Cohen’s *d* = 1.51), with lower Glutamate concentration in T2DM compared to Ctrl2 group (Fig 2C). There was no statistically significant difference in the GABA^+^_corr_/Glx_corr_ ratio between groups. Secondary analysis of other metabolites levels revealed no statistically significant differences between groups for neither tCr, mI, tCho, Glutamine nor GSH between T2DM and Ctrl2 groups. The concentration of tNAA was significantly different between groups, lower in T2DM patients (*t*_39_ = -3.10, *p* = 0.004). The observed difference in Glutamate was preserved when normalized for Creatine levels (Glutamate/tCr, *N* = 41, *Z* = -3.07, *p* = 0.002), but not for GABA^+^_corr_/tCr.

#### Glutamate is linked with the metabolic profile of type 2 diabetes patients

To model a putative effect of chronic metabolic control on the levels of GABA, Glutamate, and the I/E tonus, assessed by GABA^+^_corr_/Glx_corr_, we performed linear regression analysis within the T2DM patient’s cohort (Fig 1B–1D). Higher HbA_1c_ levels represent a worse metabolic control.

Within the T2DM group, there was a significant linear relation between HbA_1c_ levels and Glutamate (*F*(1,23) = 4.90, *p* = 0.037, *R*^2^ = 0.18), and GABA^+^_corr_/Glx_corr_ ratio (*F*(1,24) = 5.34, *p* = 0.030, *R*^2^ = 0.18), but not with GABA^+^_corr_ (*F*(1,24) = 3.76, *p* = 0.064, *R*^2^ = 0.14) nor Glx (*F*(1,24) = 1.37, *p* = 0.253, *R*^2^ = 0.05) individually. Surprisingly, there was no correlation between neither Glutamate, GABA^+^_corr_ nor GABA^+^_corr_/Glutamate ratio with PRESS-estimated Glucose levels.

**Fig 3:**
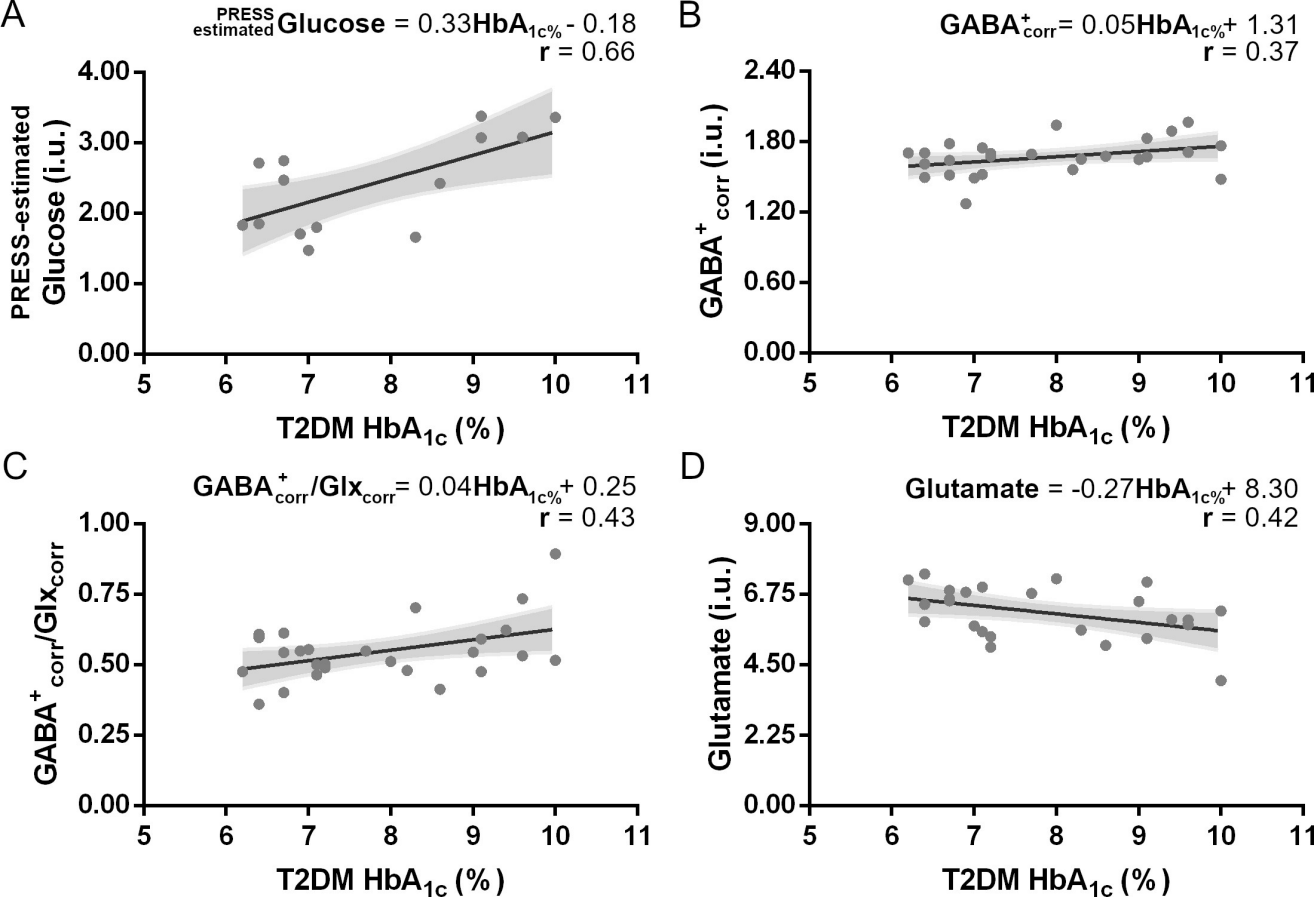
The link between neurotransmitters levels and their relationship with metabolic control in T2DM patients was assessed by regression slopes. (A) In T2DM patients, chronic metabolic control assessed by HbA_1c_ levels had a significant positive correlation with acute measures of acute glycemic status, reflected by the estimated Glucose levels from PRESS data. While in (B) it is clear that there is no significant linear relation between GABA^+^_corr_ levels and HbA_1c_, we found a significant linear relation between HbA_1c_ levels and both (C) GABA^+^_corr_/Glx_corr_ ratio and (D) Glutamate concentrations estimated by ^1^H-MRS in T2DM patients. Shaded area in the scatterplots represents the 95% CI for the best-fit line. Equations refer to the linear adjustment to data points and *r* is the linear regression coefficient of the adjustment.

Correlation analysis between HbA_1c_ levels and clinical-demographic variables showed an expected positive correlation with BMI (*N* = 25, *ρ*_*S*_ = 0.45, *p* = 0.026) but not with neither age nor disease duration. Brain Glucose levels, indirectly estimated from PRESS data, did not correlate with age, disease duration or BMI. Yet, a significant linear regression could be established with HbA_1c_ levels (*F*(1,12) = 9.28, *p* = 0.010, *R*^2^ = 0.44, Fig 3A), reflecting a close relationship between central and peripheral metabolism.

### Study B–Type 1 diabetes mellitus

#### GABA and Glutamate are balanced in type 1 diabetes

Non-parametric Mann-Whitney U tests on GABA^+^_corr_ (GABA^+^_error to water_: T1DM = 5±1.9%, Ctrl1 = 4±1.9%) and Glx_corr_ (Glx_error to water_: T1DM = 6±3.3%, Ctrl1 = 5±1.7%) concentrations showed no significant differences between T1DM and Ctrl1 groups (GABA^+^_corr_: *N* = 26, *Z* = -0.42, *p* = 0.698, Cohen’s *d* = 0.17; Glx_corr_: *N* = 25, *Z* = -0.17, *p* = 0.892, Cohen’s *d* = 0.07). As in the Study A, we isolated the Glutamate from Glutamine component of the Glx signal through PRESS data. There was no statistically significant difference in the concentration of Glutamate (CRLB: T1DM = 8±1.2%, Ctrl1 = 8±1.0%) between groups (*N* = 26, *Z* = -0.69, *p* = 0.517, Cohen’s *d* = 0.27).

**Fig 4:**
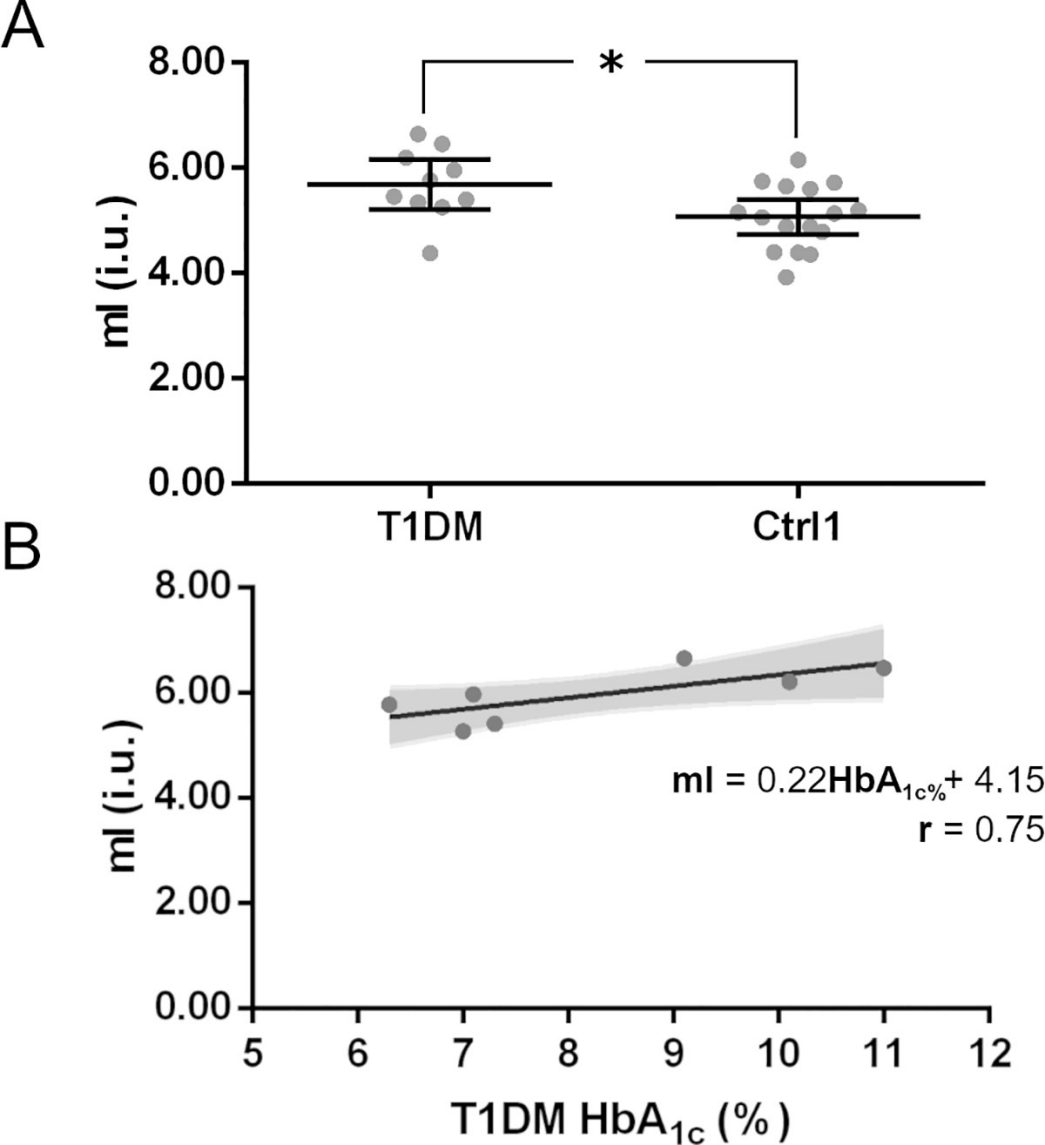
Metabolite levels in type 1 diabetes (T1DM) and age-matched healthy control (Ctrl1) groups were estimated through ^1^H-MRS. There were no significant differences on mean neurotransmitters levels between T1DM and Ctrl1 groups. However, (A) a significant statistical difference of mI levels (i.u., institutional units) between T1DM and Ctrl1 group was found. Moreover, a linear regression analysis shows (B) a positive relation between the concentration of mI (i.u.) and HbA_1c_ levels in T1DM patients suggesting possible neuroinflammation mechanisms related to chronic metabolic control. Graph (A) depict individual values and the mean (horizontal bar). Error bars represent 95% CI values. *p≤ 0.05. Shaded area in the scatterplot (B) represents the 95% CI for the best-fit line. Equation refers to the linear adjustment to data points and *r* is the linear regression coefficient of the adjustment.

Secondary analysis showed no statistically significant differences in tCr, tNAA, tCho, Glutamine and GSH. However, there was a statistically significant difference on mI levels between groups (Fig 4A), with higher levels (*N* = 26, *Z* = -2.32, *p* = 0.020, Cohen’s *d* = 1.02) in T1DM. Interestingly, this effect was also present when comparing mI/tCr between groups (*N* = 26, *Z* = -2.58, *p* = 0.009, Cohen’s *d* = 1.18). Regarding the main metabolites of interest, there were no statistical differences in neither GABA^+^/tCr, Glx/tCr nor Glutamate/tCr levels between T1DM and Ctrl1 groups.

Follow-up exploratory analysis showed a marginally significant positive relationship between HbA_1c_ levels and mI within the T1DM group (*F*(1,5) = 6.44, *p* = 0.052, *R*^2^ = 0.56, Fig 4B).

## Discussion

The pathophysiological effects of long-term glycemic disturbances in the central nervous system are still controversial. We had previously found that higher GABA levels were predictive of impaired psychophysical performance (speed and achromatic contrast discrimination) in T2DM, both at the time of evaluation and after one year [19]. However, the relation between changes in neurotransmission and chronic metabolic control remains elusive. This project aimed to evaluate key neurotransmitters changes in the brains of patients with T2DM (Study A) and with T1DM (Study B), and their relation to systemic metabolic control. We conducted two separate ^1^H-MRS studies with two independent age-matched control groups. MEGA-PRESS data allowed to quantify both GABA and Glx (Glutamate + Glutamine) using the same estimation method and PRESS data allowed to isolate Glutamate contribution. More particularly we were able to evaluate the relationship of neurotransmitters with chronic glycemic control linked to HbA_1c_ concentration.

### In T2DM, Glutamate is altered, and it is closely associated to chronic metabolic effects

Previous MRS reports in diabetes are difficult to compare and are inconclusive [16,39]. Most reports used Creatine as internal reference based on the assumption that its concentration is not affected by the disease. However here we show that this may not hold in the case of T2DM and indeed other studies have also found changes in Creatine and NAA concentrations in the brains of patients with diabetes [24,40]. This led to the need of estimating absolute concentrations of these metabolites, with water-scaling, to prevent a possible confound [30–32].

A previous study applying proton MRI (^1^H-MRS) in diabetes suggested the existence of alterations in inhibitory (GABAergic) and possibly also excitatory (indirectly assessed by the levels of the Glutamate+Glutamine pool (Glx)) neurotransmission in several brain regions [23]. However, this study was performed in a small cohort of 7 patients, with the specific complication of Diabetic Neuropathy and without specifying the type of diabetes. In contrast other studies have not replicated changes in GABAergic neurotransmission, but instead in NAA-containing compounds levels in T2DM [24,25] or in both Glutamatergic pools and NAA in type 1 diabetes [26] suggesting that neurometabolic patterns may differ across conditions and even disease states.

Our cohort of T2DM patients showed neurotransmitter changes both in terms of level and regulation (as measured by regression slopes) in the occipital cortex. We found a strong reduction in Glutamate levels and lower GABA concentration in T2DM patients compared to healthy controls. Interestingly, when investigating regression slopes, we found that only the first was negatively correlated with HbA_1c_ levels in T2DM, while GABA/Glx ratios showed a positive correlation. Glutamate content was lower in patients with worse glycemic control (with higher HbA_1c_ levels) reflecting poorer metabolic control in T2DM patients. This relationship is intriguing and may relate to distinct short and long term mechanisms regulating neurotransmitter levels [10] that will affect the I/E tonus in the brain. Moreover, the fact that neither Glutamate nor GABA were correlated to brain Glucose, indirectly estimated through MRS, suggests that Glutamate levels are mostly related to chronic metabolic control rather than acute metabolic effects in T2DM. In any case, these findings suggest the brain as a special target in T2DM, in line with the concept of central insulinoresistance [20].

As the major excitatory neurotransmitter of the CNS, Glutamate is not only critical for brain function and plasticity, but also in which concerns to mechanisms of disease such as neuro-excitotoxicity [41]. Moreover, there is a tight coupling between Glucose metabolism and synaptic activity involving the GABA-Glutamate-Glutamine cycle [8]. Accordingly, there is a linear stoichiometric proportion close to 1:1 between the fluxes of Glutamate cycling and Glucose oxidation that strongly influences the recycling of neurotransmitters and is linked to alterations in functional activity [7,42]. Likewise, synaptic Glutamate release may be a regulator for cortical Glucose consumption. In fact, the reduction in intracerebral Glutamate concentration may be related to a slower substrate flux through the Kreb’s cycle in T2DM patients.

Despite the lack of clear neurophysiological markers, there is a strong epidemiologic bond between diabetes and the development of dementia, possibly related to glycemic control and insulin dysregulation [20,21]. Recent work shows similar cortical plasticity patterns between T2DM, cognitive function and Alzheimer’s Disease (AD), including abnormal long-term potentiation (LTP)-like plasticity mechanisms and Glutamatergic neurotransmission inferred by TMS studies [43,44] and diffuse oscillatory activity slowing reflected by shifts from higher to lower frequencies in EEG power analysis [45]. Also, a subset of the ACCORD-MIND RCT trial showed that an increase of 1% in HbA_1c_ levels was associated with lower cognitive and memory test scores [46]. Additionally, several studies point towards a beneficial effect of a good long-term metabolic control on preservation of cognitive capabilities of T2DM patients, consequent from the tight association to the microvascular and neurological complications found in diabetes [11,21,22].

Importantly, the identification of correlation patterns between Glutamate, GABA/Glx ratios and HbA_1c_ levels in T2DM patients, reinforces an association between the neurotransmission and poor chronic metabolic control. Accordingly, the participants with poorer metabolic control showed lower glutamate and higher GABA levels. This is consistent with the study of Van Bussel *et al*. [47] showing that cognitive impairment in diabetes is related to higher GABA levels [47] and previous data showing that higher GABA/tCr levels were related to higher (worse) psychophysical thresholds [19] and lower brain activity as measured by the BOLD signal [48]. In fact, steady-state neurotransmitter levels may have distinct forms of biological impact (positive or negative), depending on the physiological system [49].

Our investigative framework to study the diabetic brain, showed a close relationship between abnormal neurotransmission and metabolic control in T2DM. The difference in Glutamate levels suggests a general impairment in Glutamatergic neurotransmission that plays a role as a regulatory marker of glycemic status in T2DM.

The present study has some limitations. As a trade-off for increasing SNR we acquired MRS data from a large single voxel, and our measures are indeed a pool of undistinguishable metabolic, intra- and extracellular neurotransmitter levels. Therefore, it would be interesting to perform Positron Emission Tomography (PET) with radioligands for Glutamate or GABA-receptors [50] to understand if these changes in T2DM are also present at the postsynaptic level. Non-invasive MRS and Transcranial Magnetic Stimulation (TMS) might be useful in this context [51]. To use the same estimation procedures, we also indirectly assessed excitatory neurotransmitter levels as Glx signal. However, since it is a mixed signal of Glutamine and Glutamate we opted to also analyze the later through PRESS data. Also, the macromolecule contributions to GABA signal should be accounted by using new MRS approaches with MM-suppression [52].

Future studies should address the impact of metabolic control in the I/E imbalance, if possible, at the level of synapse, preferably in animal models, to further unravel the underlying pathophysiological mechanisms. Nonetheless, our results strongly suggest that metabolic status is a relevant measure that should be carefully evaluated in functional studies.

### In T1DM neurotransmission is preserved but neuroinflammation may be present

A similar research protocol was applied in Study B. However, in T1DM, neurotransmitters levels were not different from controls. Curiously, a recent study found higher absolute Glutamate levels in participants with type 1 diabetes that correlated positively with glycemic control [53]. Despite the exploratory nature our sub-study, the interpretation of these results has some limitations due to our lower sample size that underpowers the statistical analysis. Yet, the studies are hard to compare especially due to the major differences in clinical-demographic characteristics of the cohorts, which in our case are older and with longer diabetes duration. Further research should evaluate the impact of peripheral metabolic control in T1DM in larger and more comprehensive cohorts. Follow-up analysis on other metabolites showed that instead, higher mI (and mI/tCr) levels were present in the T1DM group. A previous report showed a general increase of mI in the brains of diabetic patients and a decrease of NAA [54]. Despite a lower sample size, the regression analysis suggested a positive relationship between chronic metabolic control and mI levels in T1DM. Taken together these results suggest that neuroinflammation might dominate in T1DM in line with previous studies reporting a fluctuation in myo-Inositol in different brain areas of T1DM [54–56].

Myo-Inositol is a sugar-like molecule that acts as an osmolyte and is involved in the organization of cell membranes and myelin sheets [57]. In addition, mI is most abundant in white matter, being commonly considered as a glial biomarker. Therefore, high levels of mI may be associated with glial proliferation and membrane turnover suggesting that gliosis and neuroinflammation may be more dominant in T1DM patients.

## Conclusions

We found that disruption of systemic metabolic control is associated to changes in the neurotransmission profile (levels and regulation slopes) in type 2 diabetes. The difference in Glutamate suggests a general impairment in Glutamatergic neurotransmission in patients with T2DM. Moreover, the identified association between regulation of neurotransmitter levels and HbA_1c_ in these patients suggests a tight coupling between neurometabolism and systemic metabolic control. In general Glutamate is lower and GABA/Glx is higher when metabolic control is poor, in agreement with previous evidence showing that behavioral performance and neural responses deteriorate when relative GABA levels increase. Also, we did not find any relation between neurotransmitters levels and acute brain Glucose levels, estimated through MRS, suggesting that the variability on GABA levels is mostly related to chronic effects.

An exploratory assessment on a small cohort of T1DM patients showed no evidence for neurotransmission alterations, however the observed changes in myo-Inositol suggest an increase of membrane turnover and/or the presence of neuroinflammatory processes.

These findings support a link between abnormal neurotransmission and metabolic control in T2DM by which neurotransmission, evaluated through MRS, may reflect metabolic status. In T1DM neurometabolic processes are relatively spared, in contrast with the evidence found for inflammatory and glial activation processes.
